# A Heat and Mass Transfer Model of Peanut Convective Drying Based on a Two-Component Structure

**DOI:** 10.3390/foods12091823

**Published:** 2023-04-28

**Authors:** Pengxiao Chen, Nan Chen, Wenxue Zhu, Dianxuan Wang, Mengmeng Jiang, Chenling Qu, Yu Li, Zhuoyun Zou

**Affiliations:** School of Food and Strategic Reserves, Henan University of Technology, Zhengzhou 450001, China

**Keywords:** peanut, two-component structure, convective drying, heat and mass transfer, mathematical model

## Abstract

In order to optimize the convective drying process parameters of peanuts and to provide a theoretical basis for the scientific use of energy in the drying process, this study took single-particle peanuts as the research object and analyzed the heat and mass transfer process during convective drying. In addition, a 3D two-component moisture heat transfer model for peanuts was constructed based on the mass balance and heat balance theorem. Moreover, the changes in the internal temperature and concentration fields of peanut pods during the whole drying process were investigated by simulations using COMSOL Multiphysics. The model was validated by thin-layer drying experiments, compared with the one-component model, and combined with low-field NMR technology to further analyze the internal moisture distribution state of peanut kernel drying process. The results show that both models can effectively simulate the peanut thin-layer drying process, and consistency is found between the experimental and simulated values, with the maximum errors of 10.25%, 9.10%, and 7.60% between the simulated moisture content and the experimental values for the two-component model, peanut shell, and peanut kernel models, respectively. Free water and part of the weakly bound water was the main water lost by peanuts during the drying process, the change in oil content was small, and the bound water content was basically unchanged. The results of the study provide a theoretical basis to accurately predict the moisture content within different components of peanuts and reveal the mechanism of moisture and heat migration during the drying process of peanut pods.

## 1. Introduction

Peanut (*Arachis hypogaea* L.), also known as groundnut, originated in the tropical and subtropical regions of South America, and is widely grown in China, India, the United States, and Argentina, thereby making it one of the world’s most important cash crops [1,2,3,4]. In addition, its production ranks second in the world among legumes, following soybeans [5]. It has a protein content of 16–36% and fat content of approximately 50%, and is also rich in a range of nutrients, including unsaturated fatty acids, vitamin E, sterols, and phenolic compounds [6,7]. Freshly harvested peanuts have a moisture content of up to 50% [8], but they are highly susceptible to mold and even toxins if they are not dried in time, thereby resulting in economic losses [9,10,11]. Peanuts with less than 10% moisture can be stored for a long time [12]. With 9% moisture content, it can be stored safely for nearly 1 year at 25–27 °C and 70% RH [13]. Therefore, drying is one of the most important aspects to guarantee the quality of peanuts after production [14,15,16]. The drying technologies applied to nuts mainly include natural drying, convective drying, microwave drying, vacuum drying, etc. Currently, combined drying technologies such as pulsed vacuum drying (PVD) [17], radio frequency combined convective drying (RF-HAD) [18], and hybrid infrared-hot air (IR-HAD) [19]—which not only improve the drying efficiency but also the quality of the product—comprise the current research hot spot. However, combined drying technology is not yet mature; convective drying is still the most widely used drying method for peanuts because of its advantages such as large processing capacity and easy control.

Moreover, drying is a complex heat and mass transfer process in which the temperature and moisture content of the material is constantly changing under the combined effect of temperature and moisture gradients [20]. The drying rate is influenced by the physical structure and composition of the material. Peanut pods are non-homogeneous bodies with complex geometric structure, consisting of two parts—peanut shell and peanut kernel—and the initial moisture distribution is not uniform. Studying it as a homogeneous body differs greatly from the actual situation; it cannot accurately account for its internal moisture and heat transfer mechanism. Using mathematical models to study the drying heat and mass transfer process is a low-cost method for process optimization which has been widely used in recent years [21,22,23,24]. Multi-component models are more accurate than single-component models [25]. Huang et al. [26] developed separate mathematical models for drying maize kernels to study the internal moisture changes, thereby assuming that the kernels consist of single-component homogeneous and multi-component inhomogeneous bodies. By comparing COMSOL Multiphysics simulation with thin-layer drying experiments, the simulation accuracy of the multi-component model was better than that of the single-component model. Takhar et al. [27,28] reconstructed a 3D four-component geometric model of maize seeds using CT scanning technology, and the model accuracy was further improved by simulating the stress distribution as well as moisture content changes of each component of maize seeds during convective drying. Zhao et al. [29] constructed a 3D geometric model of rice grain using image processing and simulated its hot air-drying process using COMSOL Multiphysics software. The simulation and experimental results showed that hot air temperature was one of the main factors affecting the drying process of rice grain. Ghosh et al. [30] obtained the moisture content and temperature distribution of different components of wheat seeds during drying by constructing a 3D two-component geometric model of wheat seeds combined with a mathematical model of wet heat transfer. However, at present, only a few studies have been conducted on the drying process by building a 3D two-component solid model of peanut pods.

Convective drying is a common drying method in peanut production [31]; however, its drying efficiency is relatively low [32]; hence, the wet heat transfer mechanism still needs to be further investigated. In this study, a 3D two-component wet heat transfer model for single-particle peanuts was constructed based on the principle of thermal mass balance. Considering the inhomogeneity of the initial moisture content distribution of different components, the temperature distribution and variation of the peanut convective drying process was simulated, the mechanism of moisture migration during the drying of peanut pods was studied, and the model was validated through drying experiments, thereby aiming to provide a theoretical basis for the optimization of the peanut convective drying process and the scientific use of energy in the drying process. 

## 2. Materials and Methods

### 2.1. Materials

This study adopted fresh peanuts produced in Kaifeng City, Henan Province, and the variety was Kainong 308. Before the experiment, peanuts with complete pods were selected—consistently and full sized, with no wormholes—and placed in a self-sealing bag and stored in a 4 °C refrigerator for standby. The initial dry basis moisture content of peanut pods, shells, and kernels measured by drying at 105 °C to constant mass was 0.83 ± 0.03 g/g, 1.24 ± 0.02 g/g, and 0.74 ± 0.03 g/g, respectively.

### 2.2. Equipment

**Figure 1:**
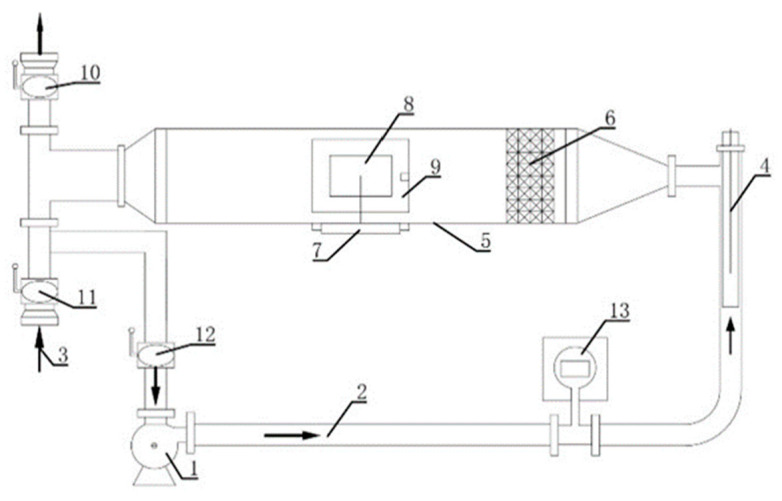
Schematic diagram of the customized digital cavity experimental facility. (The arrows represented the wind direction; 1. Blower; 2. Pipeline; 3. Air inlet; 4. Heater; 5. Drying room; 6. Airflow distributor; 7. Weighing sensor; 8. Materials; 9. Inlet and outlet hatch; 10., 11., 12. Butterfly valve; 13. Gas flow meter).

The customized digital cavity drying experiment device (Figure 1) was purchased from Xiangtan Tianke Instrument Co., Ltd. (Xiangtan, China). The DHG-9076A electric constant temperature blast drying oven and FA1004 analytical balance were purchased from Jintan Shenglan Instrument Manufacturing Co., Ltd. (Jintan, China) and Shanghai Hengping Instrument Factory (Shanghai, China). The Hot Disk TPS2500S thermal constant analyzer was purchased from Hot Disk, Göteborg, Sweden.

### 2.3. Determination of Thermal Conductivity and Specific Heat Capacity

The drying characteristics and their laws depend not only on the external drying conditions but are also influenced by the physical parameters of the material itself. The transient planar heat source (TPS) method is currently a more advanced method to measure the thermal physical parameters of materials developed based on the transient tropical method and the transient hot wire method [33]. Thus, to obtain accurate values of the model parameters, this paper uses a Hot Disk TPS2500S thermal constant analyzer with a 5501 probe at room temperature to determine the moisture content of peanut shells (40%, 37%, 34%, 31%, 28%, 25%, 22%, 19%, 16%, 13%, and 10%) and peanut kernels (40%, 34%, 28%, and 22%). The thermal conductivity and specific heat capacity of peanut shells (40%, 34%, 34%, 31%, 28%, 25%, 22%, 28%, 22%, 16%, 13%, and 10%) and peanut kernels (40%, 34%, 28%, 22%, 16%, and 10%) were averaged by repeating each set of experiments thrice and then fitted to the equations of the model parameters concerning moisture content.

### 2.4. The Thin-Layer Drying Experiment

The following drying conditions that could better preserve the quality of peanuts were selected based on the previous experiment [34]: Air temperature, air speed, and air relative humidity of 42 °C, 0.75 m/s, and 8 ± 2%, respectively. The drying endpoint was set at 0.10 (d.b.), and the drying curves of peanut pods, shells, and kernels were measured separately. The drying experiments were repeated thrice under the same experimental conditions and the experiment data were averaged. During drying, the moisture content was weighed at intervals and the drying curves were plotted. The dry basis moisture content was calculated based on Equation (1).

(1) $$W_{t}=\frac{m_{t}-m}{m},$$

where $m_{t}\;$ is the mass dried to any moment *t* (g), and *m* is the mass of dry matter (g).

### 2.5. Low-Field NMR Moisture Determination

The peanut kernels in the drying process were taken, cut into small rectangles, weighed 1 g into the sample tube, placed in a variable temperature NMR food agricultural imaging analyzer for the determination of the transverse relaxation time T_2_ of the samples, and the average value was taken by repeating the measurement twice.

The parameters were set: the main magnetic field strength was 0.51 T, the sample chamber temperature was 35 °C. The CPMG pulse sequence parameters were as follows: the main frequency (SF1) was 20 MHz, the offset frequency (O1) was 998,922.4 Hz, the 490° pulse time (P1) was 3 μs, the 180° pulse time (P2) was 6 μs, the number of sampling points (TD) was 360,000, the number of echoes (NECH) was 18,000, the echo time (TE) was 0.1 ms, the sampling interval (TW) was 2500 ms, the accumulation number (NS) was 32, the analog gain (RG1) was 20 db, the digital gain (DRG1) was 3, and the repetition time is 10,000 ms.

### 2.6. The Thin-Layer Drying Simulation

#### 2.6.1. Assumptions

1. The initial moisture and temperature within the different components of peanuts were uniformly distributed;
2. The evaporation of water occurs inside the peanut and the energy effects accompanying the surface were not taken into account;
3. The shrinkage of peanuts during drying was not taken into account;
4. The temperature, relative humidity, and velocity of hot air around peanuts were kept constant during drying;
5. All components of the peanuts were isotropic and homogeneous;
6. The calculation of the moisture diffusion coefficient was based on the infinite slab shape assumption model.

#### 2.6.2. Geometric Model

**Figure 2:**
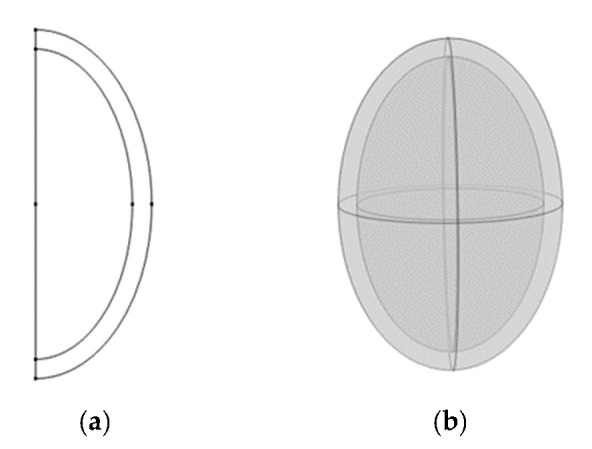
The 2D axisymmetric geometric model of the peanut (**a**); the 3D two-component structure of the peanut (**b**).

The gap between peanut shell and kernel was neglected to simplify the calculation process, and the 3D two-component structure of the peanut was simplified by making a 2D axisymmetric treatment to obtain an ellipse with an a- and b-axis of 5 mm and 8 mm (Figure 2a), respectively, nested in a shell of 1 mm thickness (Figure 2b). During the simulation, the cross-section was set to a symmetric boundary. The geometric model was meshed using a hyper-detailed free-dissecting triangular mesh, with each component obtaining a different number of mesh triangles and vertices (Table 1). Single-component simulations were performed with the same dimensions as the same individual component within multiple components.

#### 2.6.3. Heat Transport Equation

(2) $$\nabla \left(k_{i}\cdot \nabla T\right)=\rho c_{p}\frac{\partial T}{\partial t}-\rho h_{g}\frac{\partial M}{\partial t},$$

where *k_i_* (W/(m K)), *T* (K), *M* (d.b.), ∇, *t* (s), *c_p_* (J/(kg K)), *ρ*(kg/m^3^), *h_g_* (J/kg) represent thermal conductivity of peanuts, temperature, moisture content, divergence operator, time, specific heat of peanuts, density of peanuts, and latent heat of vaporization, respectively.

Initial and boundary conditions:

(3) $${\left\{\begin{array}{c} \;\;{\left.T\right|}_{t=0}=T_{0}\;\;\;\;\;\\ -k_{i}\frac{\partial T}{\partial t}=h_{T}\left(T-T_{a}\right) \end{array}\right.}_{,}$$

where *T*_0_ (K), *T_a_* (K), and *h_T_* (W/(m^2^·K)) denote the initial temperature of peanuts, temperature of the drying medium, and convective heat transfer coefficient, respectively.

The convective heat transfer coefficient (*h_T_*) is estimated using the Nusselt number (*Nu*) [35].

(4) $$\left\{\begin{array}{c} Nu=\frac{h_{T}d}{k_{a}}=2+0.552Re^{0.53}Pr^{\frac{1}{3}}\\ Re=\frac{v_{a}\rho_{a}d}{\mu_{a}}\\ Pr=\frac{\mu_{a}c_{a}}{k_{a}} \end{array}\right.,$$

where *d* (m), *k_a_* (W/(m·K)), *Re,* and *Pr* represent the equivalent diameter of peanuts, thermal conductivity of the drying medium, Reynolds number, and Prandtl number, respectively. In addition, *v_a_* (m/s), ρ*_a_* (kg/m^3^), μ*_a_* (Pa·s), and *c_a_* (J/(kg·K)) denote the velocity of the drying medium, density of the drying medium, dynamic viscosity of the drying medium, and specific heat of the drying medium, respectively.

#### 2.6.4. Moisture Transport Equation

Initial and boundary conditions:

(5) $$\left\{\begin{array}{c} \;\;\;{\left.M\right|}_{t=0}=M_{0}\\ -D_{i}\frac{\partial M}{\partial t}=h_{m}\left(M-M_{e}\right) \end{array}\right.,$$

where *h_m_* (m/s), *M_i_* (g/g), *M_e_* (g/g), and *D_i_* (m^2^/s) represent the convective moisture transfer coefficient of peanuts, initial moisture content of different components in peanuts, equilibrium moisture content of peanuts, and moisture diffusion coefficients of different components in peanuts, respectively. Meanwhile, *i* = 0, 1, and 2 denote peanuts, shells, and kernels, respectively.

The convective mass transfer coefficient (*h_m_*) was estimated using the Sherwood number (*Sh*) [35].

(6) $$\left\{\begin{array}{c} Sh=\frac{h_{m}d}{D_{a}}=2.0+0.552Re^{1/2}Sc^{1/3}\\ Sc=\frac{\mu_{a}}{\rho_{a}D_{a}} \end{array}\right.,$$

where *D_a_* (m^2^/s) and *S_c_* represent the diffusion coefficient of moisture in hot air and Schmidt number, respectively.

#### 2.6.5. Effective Diffusion Coefficient of Moisture

The effective diffusion coefficient of moisture determines the mass transfer capacity of moisture within the material and is not only related to the material composition, moisture content, porosity, and other physical parameters, but also depends on drying conditions and method [20]. Therefore, the values are measured by drying experiments and calculated by substituting them into a mathematical model to obtain a more accurate effective diffusion coefficient of moisture. The control mechanism of unsteady Fick’s second law for drying is the case of moisture diffusion control. It is assumed that the diffusion coefficient is constant, and the shrinkage of materials is ignored during the drying process [36,37]. The effective diffusion coefficient of moisture at this temperature is calculated by plotting the drying curve from a drying experiment based on the principle of the inverse method [38].

(7) $$MR=\frac{M_{t}-M_{e}}{M_{0}-M_{e}}=\frac{8}{\pi^{2}}\sum_{n=0}^{\infty }\frac{1}{{\left(2n+1\right)}^{2}}exp\left(\frac{-{\left(2n+1\right)}^{2}\pi^{2}D_{eff}t}{4L^{2}}\right)$$

In this experiment, the drying time is longer, which is simplified to:

(8) $$R=\frac{M_{t}}{M_{0}}=\frac{8}{\pi^{2}}exp\left(-\frac{\pi^{2}D_{eff}t}{4L^{2}}\right).$$

The logarithmic transformation on both ends of the above formula is calculated to obtain the following formula:

(9) $$lnMR=ln\frac{8}{\pi^{2}}-\frac{D_{eff}\pi^{2}t}{4L^{2}},$$

where *D_eff_* (m^2^/s), *L* (m), *MR*, and *t* (s) represent the moisture diffusion coefficients of peanuts, the thickness of the peanut, moisture ratio, and drying time, respectively.

As shown in Equation (9), the effective moisture diffusion coefficient can be calculated from the slope of the curve of *lnMR* versus drying time *t*. The effective moisture diffusion coefficients of peanut pods, shells, and kernels were 5.7512 × 10^−10^ m^2^/s, 1.63249 × 10^−10^ m^2^/s, and 2.91731 × 10^−10^ m^2^/s, respectively.

#### 2.6.6. Model Parameters

The above parameters are not only used in the specific numerical simulations but also in some other main model parameters, as shown in Table 2.

#### 2.6.7. Numerical Analysis

The COMSOL Multiphysics software (version 6.0) was used in this paper to solve the above transient heat and mass transfer equations using the selected solid heat transfer module and dilute matter transfer module. The solution process started with a mesh verification to determine the mesh size. The time step was set to 1 min and the termination time to 1230 min. The simulation was carried out on a Dell 5820 computer workstation (Windows 10 64-bit operating system, running memory 32 GB) with 4889 degrees of freedom (plus 4490 internal degrees of freedom), and the overall solution time was approximately 59 s.

## 3. Results and Discussion

### 3.1. Thermal Conductivity and Specific Heat Capacity

**Figure 3:**
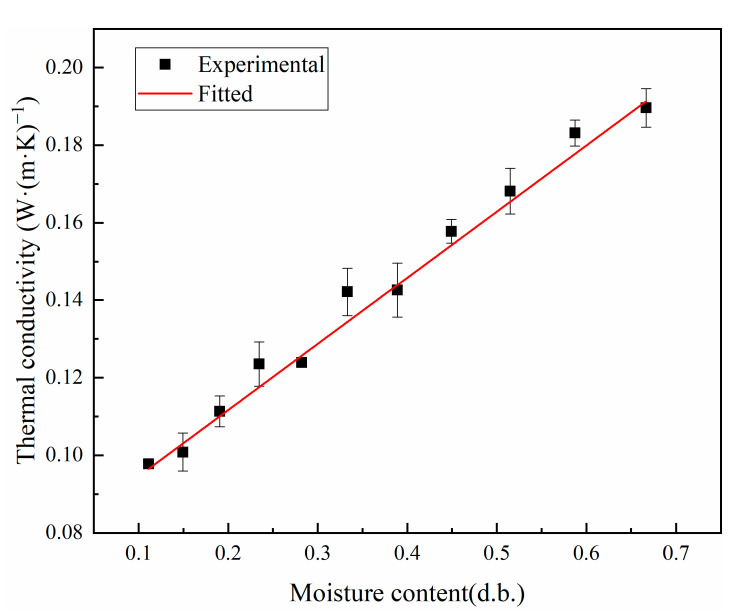
Thermal conductivity of shells at different moisture contents.

Figure 3 shows a plot of the experimentally measured thermal conductivity of shells at different moisture contents, wherein a linear relationship is observed between the two. By fitting the experimental data, the linear function relationship was obtained as follows:

(10) $$k_{1}=0.17062M+0.07753,$$

where *M* is the dry basis moisture content of peanut shells, g/g; correlation coefficient *R*^2^ = 0.98973.

**Figure 4:**
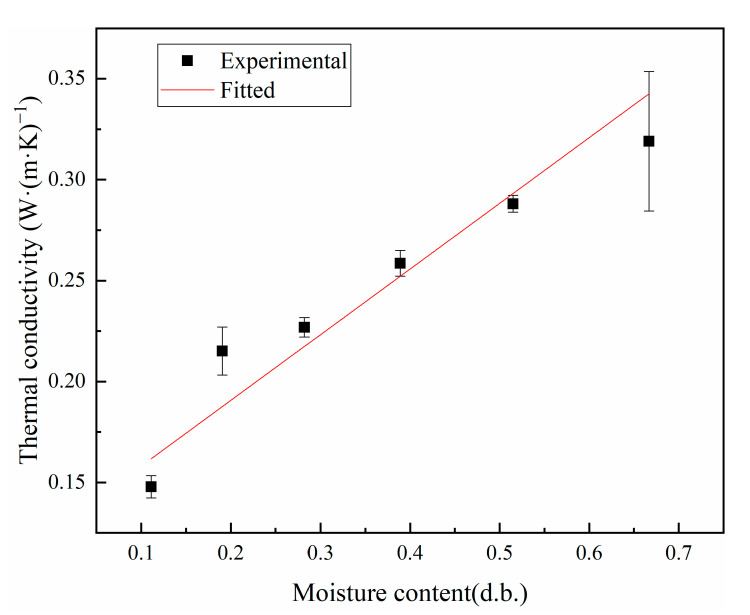
Thermal conductivity of kernels at different moisture contents.

Figure 4 shows a plot of the experimentally measured thermal conductivity of kernels at different moisture contents, wherein a linear relationship is found between the two. By fitting the experimental data, the linear functional relationship was obtained as follows:

(11) $$k_{2}=0.32528M+0.12559,$$

where *M* is the dry basis moisture content of peanut kernels, g/g; correlation coefficient R^2^ = 0.95737.

The thermal conductivity increases linearly with the increasing moisture content of the dry base, with peanut shells having a lower thermal conductivity than peanut kernels. This is because the thermal conductivity of the samples was lower than that of water and higher than that of hot air, and the experiments experimented with the combined thermal conductivity of the sample and air mixture [41]. For the initial sample, the thermal conductivity is much lower than that of water and has good hygroscopic properties; hence, it is susceptible to the effect of water content and shows the same trend as the water content. As the water content gradually increases, the proportion of air between the pores of the sample gradually decreases and the thermal conductivity also gradually increases. The peanut shell is a fibrous tissue with larger pores, the bulk weight of the peanut kernel is higher than that of the peanut shell, and the porosity decreases with increasing bulk weight, thereby resulting in a lower porosity of the peanut kernel than that of the peanut shell. Therefore, the thermal conductivity of the peanut kernel is higher than that of the peanut shell.

**Figure 5:**
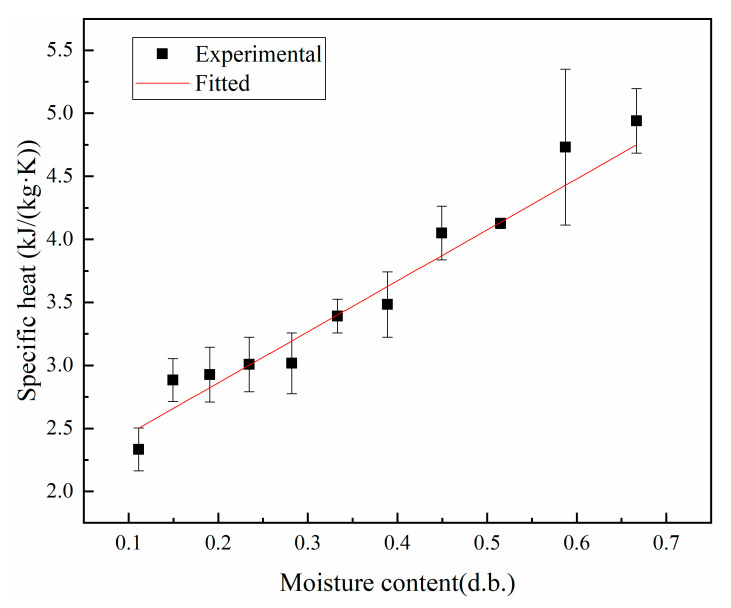
Specific heat of shells at different moisture contents.

Figure 5 shows a plot of the experimentally measured specific heat of shells at different moisture contents, wherein a linear relationship is observed between the two. By fitting the experimental data, the linear functional relationship was obtained as follows:

(12) $$c_{1}=4.04506M+2.05226,$$

where *M* is the dry basis moisture content of peanut shells, g/g; correlation coefficient R^2^ = 0.97736.

**Figure 6:**
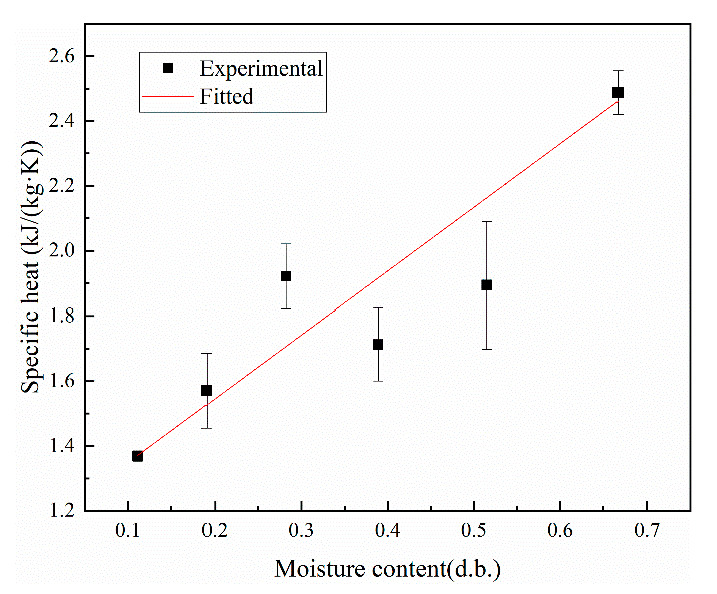
Specific heat of kernels at different moisture contents.

Figure 6 shows a plot of the experimentally measured specific heat of kernels at different moisture contents, wherein a linear relationship is found between the two. By fitting the experimental data, the linear functional relationship was obtained as follows:

(13) $$c_{2}=1.96152M+1.15307,$$

where *M* is the dry basis moisture content of peanut kernels, g/g; correlation coefficient R^2^ = 0.96583.

The specific heat capacity increased with the increase in moisture content of the wet base. This is because the specific heat capacity of the sample is higher than that of hot air and lower than that of water. As the water content of the sample increases, the proportion of water in the air-filled pores continues to increase, thereby resulting in a change in the composition of the sample with air and water. As the water content increases, the proportion of air gradually decreases; hence, the specific heat capacity increases linearly [42]. In particular, the specific heat capacity of peanut shells was higher than that of peanut kernels, which may be due to the different chemical composition of the two; the main components of peanut kernels are denser proteins and fats, but the other is crude fiber, which has higher hygroscopic properties. The differences in the thermal physical parameters of the two components caused differences in the respective heating rates.

### 3.2. Verification and Simulation of Peanuts during Drying

**Figure 7:**
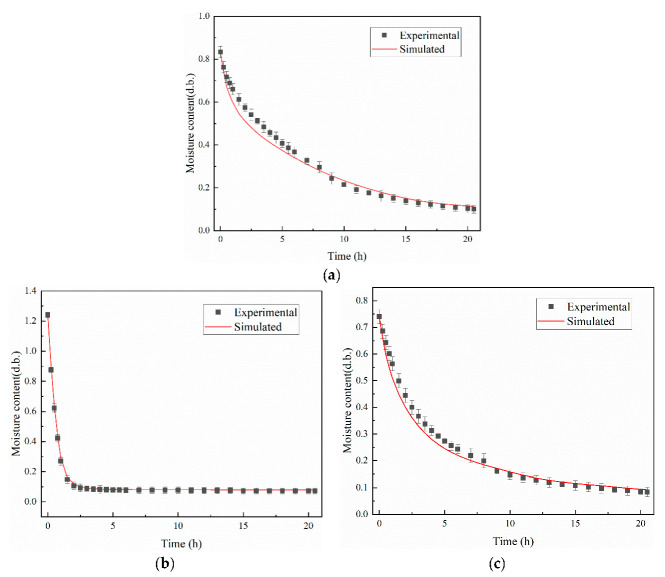
Comparison of simulated and experimental average water content of peanut pods during drying (**a**); comparison of simulated and experimental average moisture content of peanut shells during drying (**b**); comparison of simulated and experimental average moisture content of peanut kernels during drying (**c**).

Figure 7 shows the experimental and simulated results of the dry basis moisture content of peanut pods, shells, and kernels during thin-layer drying at 42 °C (hot air temperature). The trends of the simulated and experimental values were consistent. As shown in Figure 7a, the simulated values underestimated the moisture content of peanut pods in the early stage of drying, and the difference between the two gradually decreased as the drying progressed when the peanut pods were simulated as a whole in a two-component simulation. At the end of drying, the simulated value was higher than the experimental value, and the difference between the two gradually increased again. Besides the initial warming process, the peanut pods showed typical falling rate drying characteristics. The total drying time was 20.5 h. The rate of moisture content reduction was 3.58%/h for the thin-layer drying experiment and 3.53%/h for the simulation, with a maximum relative error of 10.25% throughout the drying process. This indicated that the model can be used to further investigate the changes in temperature and moisture content during the drying of peanut pods. Not only did the moisture gradient affects moisture diffusion, but the difference in physical structure and chemical composition of the two also contributed to the differences. In the early stages of drying, peanut shells and kernels were less porous, and as drying proceeded, the structure was gradually deformed, and the porosity continued to increase. The peanut pods also produced shrinkage deformation during drying, thereby resulting in a shorter distance of water migration within the peanut pods, thus accelerating the rate of water migration. In addition, at the end of the drying period, peanut pods were cracked under long and continuous high-temperature drying conditions, which exposed the interior to air, thereby reducing the skin resistance and increasing the contact area between the interior of the peanut pods and the hot air, thus accelerating the moisture migration rate [26]. However, the model did not take into account the shrinkage deformation, cracking, and porosity changes of peanut pods in the simulations, which resulted in errors between the experimental and simulated values.

Figure 7b,c shows the single-component simulation of peanut shells and kernels as two separate parts. The moisture content of peanut shells and kernels was less than 0.1 (d.b.) after 2.5 h and 17 h drying, respectively. After 20.5 h of drying, the rates of moisture content reduction of peanut shells during the thin-layer drying experiment and simulation were 5.71%/h and 5.67%/h, and the rates of moisture content reduction of peanut kernels during the thin-layer drying experiment and simulation were 3.20%/h and 3.16%/h, respectively. Among them, when drying for 2 h, the difference between the simulated value of the peanut shell and the experimental value is the largest, with a relative error of 9.10%. Meanwhile, when drying for 4 h, the simulated value of the peanut kernel and the experimental value have the largest difference, with a relative error of 7.60%. The maximum relative error of both is lower than the relative error of the two-component model, which may be due to the complex diffusion mechanism of water transfer from a peanut kernel to a peanut shell. During the early stage of drying, the peanut shells acted as a protective barrier before the peanut kernel came into contact with hot air, and the internal moisture gradually migrated outward, with a faster drying rate and structural contraction occurring before the peanut kernel. As drying proceeds, the internal temperature of the peanut kernel gradually rises, and the rate of contraction accelerates. In addition, given that the physical structure and composition of peanut shells and kernels are different, the degree of shrinkage and deformation is also different. The main components of peanut kernels are protein and fat, and their mesh structure is prone to collapse when the cells enter a dehydrated state, whereas peanut kernels are thicker and more deformable. Meanwhile, the main component of peanut shells is cellulose, large channels, and stronger moisture absorption, and its good mesh structure is not easily affected by changes in moisture content, as well as by deformation. The asynchronous shrinkage of the two and the difference in shrinkage ratios lead to a gradual increase in the void between peanut shells and kernels. Furthermore, their air layer has an obstructive effect on the migration of moisture between peanut shells and kernels, thereby resulting in a difference in pressure gradient between the inside and the outside and increasing the migration path of peanut kernel moisture [43]. Peanut kernel moisture diffused outward through the peanut shell, thereby affecting the drying efficiency of peanut shells.

Peanut shells and kernels were simulated separately as a single component, and the simulation accuracy of both was slightly higher than that of the two-component model. The error was perhaps due to the differences between the actual material and the parameters assumed in the simulation. Moreover, the model does not take into account shrinkage, voids between shells and kernels, variations in porosity, and a simplified treatment of the physical geometry model. However, in practical applications, peanut pods were often dried as a whole. Therefore, exploring the complex diffusion mechanism of moisture transfer from peanut kernel to peanut shell and continuing to optimize the model can be used to study the two-component moisture gradient variation in peanuts with more accuracy.

In addition, the drying curve of peanut shells and kernel changes trends greatly differ. Peanut shell structure contains larger pores of fibrous tissue, whereas peanut kernel structure is denser. In the early stage of drying, peanut kernel porosity changes are not evident, while there is a mass transfer resistance in peanut kernel moisture migration. At this time, the drying object is primarily the shell, which led to a rapid decline in the moisture content of the peanut shell. In the middle of drying, peanut kernel water loss, and porosity changes accelerated; hence, the peanut kernel moisture decline rate accelerated. At this time, the peanut shell structure changed more slowly, and the mass accounted for is relatively small, thereby limiting its moisture migration rate. Therefore, the drying process between the two components of peanut pods greatly differed, and the division of this drying process into two parts was conducive to the continued investigation of the mechanism of moisture and heat transfer in the drying process of peanut pods.

### 3.3. Changes in the State of Moisture Distribution during Drying

**Figure 8:**
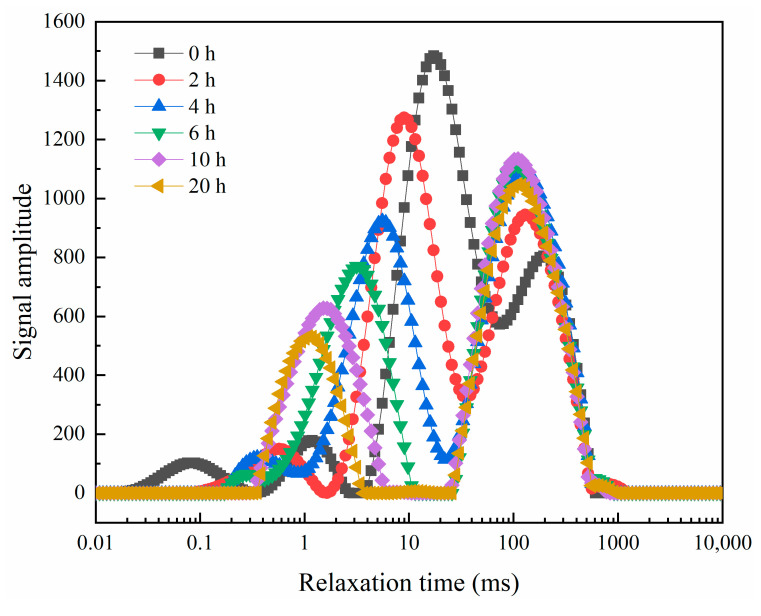
NMR transverse relaxation spectra of peanut kernel during drying.

The transverse relaxation time T_2_ inversion profiles of peanut kernels during drying are shown in Figure 8, and there are mainly four relaxation peaks with relaxation times of 0.1, 1, 10, and 100 ms corresponding to the relaxation peaks of bound water T_21_, weakly bound water T_22_, free water T_23_, and oil T_24_, respectively [44]. In the early stage of drying, the free water content was high, and the bound water and weakly bound water accounted for a smaller percentage. Due to the close relaxation time, the free water and oil peaks overlapped. As the drying proceeded, the T_23_ peak area changed more, i.e., the free water content decreased faster, the T_22_ peak gradually disappeared and the whole wave shifted to the left, and the T_23_ and T_24_ peaks gradually separated. Due to the decrease of free water content, the T_24_ peak increased a relatively small amount. In the later stages of drying, weakly bound water and most of the free water were removed. The T_23_ peak decreased slightly, which may be due to the fact that convective drying lasting for a longer period of time causes a decrease in fat content [45]. Thus, free water and a part of the weakly bound water comprise the main water dissipated from peanuts during drying, with small changes in oil content and essentially unchanged bound water content. Drying makes the internal structure of peanut kernels deformed, and the binding force of weakly bound water is reduced, which is then partially converted into free water to be removed; while bound water is more stable and tightly bound to proteins and other macromolecules through hydrogen bonding, which is not easily removed [46].

### 3.4. Variation of Moisture and Temperature Gradient of Peanut during Drying

#### 3.4.1. Moisture Gradient

**Figure 9:**
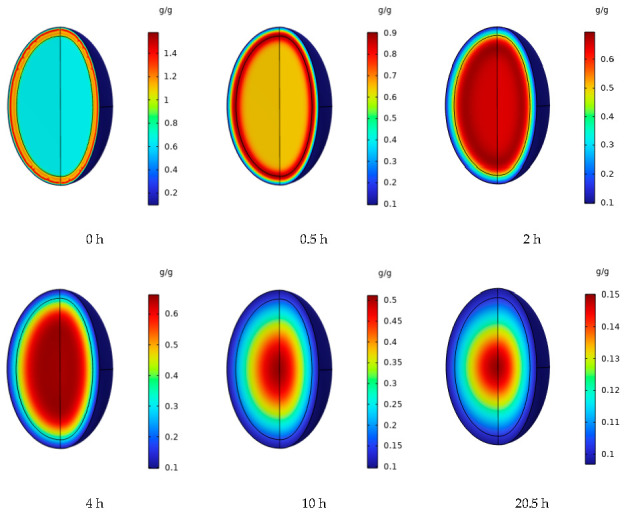
Changes in the internal moisture gradient of peanuts during drying.

Figure 9 shows the changes in the internal moisture distribution of peanuts at different moments during the drying process, wherein the internal moisture distribution of peanuts was more uniform at the initial moment, and the moisture content near the epidermal boundary layer was lower. After 0.5 h of drying, the internal moisture gradually migrates outward, a moisture gradient appears inside the peanut shell, and the peanut kernel has a high moisture content. Then, 2 h later, the peanut shell initially reached safe moisture, with an average dry basis moisture content of 0.1063, at which time the moisture content of the peanut kernel only slightly changed. At the early stage of drying, the internal moisture of the peanut continuously diffused to the surface of peanut shells in the form of convection for wet heat transfer [47], which was faster as compared to the transfer rate of the internal moisture diffusion form of the peanut kernel [48]. Subsequently, 4 h later, the moisture difference between the two components decreased, and the average dry basis moisture content of the peanut kernel was 0.3131, and the drying rate gradually decreased from 4 h to 10 h. This is because of the existence of free and bound water within peanuts. Free water in the free state has high mobility and is the easiest to remove first; meanwhile, combined water and protein (as well as other materials) is difficult to remove. At the early stage of drying, most of the free water inside the peanut rapidly evaporates, thereby resulting in a rapid decline in water content. The rate of diffusion of bound water and the rate of migration to the surface are low and slow, respectively. The diffusion coefficient gradually decreases, thereby leading to a gradual decrease in the drying rate, which is similar to the results of Wei et al. [25].

As drying proceeded, the difference between internal and surface moisture of peanuts gradually decreased. ElGamal et al. [49] reported a similar result using a rice model. As the drying proceeds, the moisture content of the peanut kernel gradually decreases from the center to the edge, the moisture gradually spreads outward, and the moisture content gradually decreases. From the perspective of heat and mass transfer, hot air drying is convective drying, and this is the heating process from the outside to the inside. The closer to the center, the more sluggish the diffusion of moisture migration. The difference in the moisture gradient can be attributed to the mass transfer and corresponding surface exchange [50]. During the initial stage of drying, it is primarily carried out on the surface of materials, and heat and mass exchange is primarily carried out through surface moisture vaporization. The loss of surface water leads to a moisture gradient inside the material, thereby resulting in a gradually decreasing trend of water distribution from the center to the edge. After 20 h of drying, the moisture content of peanut kernels dropped to a low level, with an average dry basis moisture content of 0.0992 at 20.5 h, thereby meeting the safe storage moisture requirements for peanuts (<0.1 d.b.).

#### 3.4.2. Temperature Gradient

**Figure 10:**
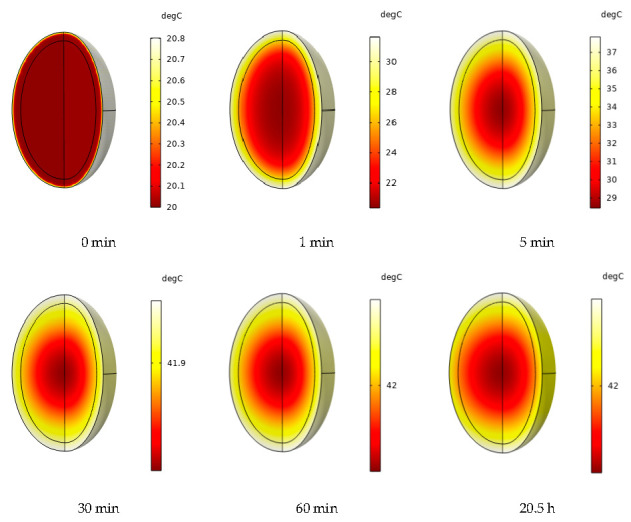
Changes in the internal temperature gradient of peanuts during drying.

Simulation of the hot air-drying process, the initial temperature of 20 °C, hot air speed of 0.75 m/s, and peanuts in different moments of the internal temperature distribution changes are shown in Figure 10. The temperature distribution inside the peanut was uniform at 0 min, and there was no heat transfer at this time. With the drying process, the peanut internal temperature gradient. At 1 min, when the peanut kernel which can be seen in the center of the temperature is low, then the peanut shell temperature becomes high, and the surface temperature quickly rises to approximately 30 °C. Onwude et al. [51] also observed similar behavior of sweet potato samples during convective drying. This is because of the drying of peanuts from the outside to the inside of the heating process. The drying of the early peanut shell temperature gradient is larger and the peanut kernel temperature gradient is smaller; that is, the gradient of heat change is from the outside to the inside. As drying proceeds, the internal temperature rises gradually. At 5 min, the internal temperature distribution of peanuts tends to be uniform, the central temperature tends to be close to the surface temperature, and the temperature difference is further reduced. At this time, the surface temperature has not reached the hot air temperature, and peanuts continue to heat. Then, at 5–30 min, between the temperature field, changes tend to slow, and the internal temperature is close to the external air temperature. At 30–60 min, the temperature difference is only about 0.1 °C, reaching a preset temperature of 42 °C. After the temperature field reaches a steady state, the temperature no longer changes. The temperature gradient inside the peanut pods was more pronounced at the initial stage of drying, which is consistent with the findings of Sabarez et al. [52]. On the one hand, this is because heat transfer is from the outside to the inside, contrary to the moisture transfer process acting first on the exterior. On the other hand, peanut shells are fibrous tissues with high porosity, the heat diffusion rate of peanut shells is greater than that of peanut kernels at the beginning of drying, and the temperature can be transferred to the interior more quickly. In addition, the thermal conductivity and specific heat capacity are dependent on moisture, and changes in moisture concentration caused by moisture transfer further affect the temperature field through heat loss because of evaporation and changes in the thermal conductivity and specific heat capacity of the peanut shells and kernels. Therefore, in convective drying, drying is dominated by internal diffusion [53]; that is, the mass transfer process controls the convective drying process.

## 4. Conclusions

(1) The results of the simulation study and the thin-layer drying experiment showed that the peanut 3D two-component moisture heat transfer model had high accuracy. The maximum error between the simulated and experimental values of the dry basis moisture content of two-component peanut pods during drying was less than 10.25%. In addition, the maximum errors between the simulated and experimental values of the moisture content of peanut shell and peanut kernel models were 9.10% and 7.60%, respectively. Therefore, the numerical model proposed in this paper can successfully predict the drying process of peanut pods with two components under hot air-drying conditions.

(2) The results of modeling the temperature and concentration fields revealed that heat was primarily transferred inward by convection from the air to the surface of the material and by conduction from the surface to the center of the material, while moisture diffused outward to the surface of the material and evaporated. The temperature gradient existed for a shorter period within the peanut as compared to the moisture gradient; therefore, the moisture heat transfer during drying was dominated by mass transfer processes.

(3) The drying characteristics were not only dependent on the external drying conditions but were also influenced by the physical parameters of the sample. The thermal conductivity became larger with the increasing moisture content during the drying process and showed a single linear relationship with the moisture content; the specific heat capacity was composed of both solid skeleton and moisture, but primarily dominated by moisture. The lower the moisture content, the smaller the specific heat capacity.

(4) The results of the low-field NMR study showed that free water and a portion of the weakly bound water were the main water types dissipated from peanut kernels during the drying process, with small changes in oil content and essentially unchanged bound water content.

## Figures and Tables

**Table 1:** Area and elements of the two-component in the peanut geometry of the 1/2 section grid.

	Shells	Kernels
Mesh vertex	319	742
Triangle	506	1378
Grid area	21.99 mm^2^	62.8 mm^2^

**Table 2:** Physical parameters of peanuts and hot air.

Parameters	Values or Expressions	References
Temperature of hot air $T_{a}$/(°C)	42	Measured
Initial temperature $T_{0}$/(°C)	20	Measured
Density of peanut shell $\rho_{1}$/(kg/m³)	560	Measured
Density of peanut kernel $\rho_{2}$/(kg/m³)	1000	Measured
Density of hot air $\rho_{a}$/(kg/m^3^)	8.666 × 10^−6^*T_a_*^2^ − 4.318 × 10^−3^*T_a_* + 1.288	[39]
Specific heat capacity of hot air *c_a_*/(J/(kg·K))	4.834 × 10^−4^*T_a_*^2^ − 2.218 × 10^−2^*T_a_* + 1007	[39]
Thermal conductivity of hot air *k_a_*/(W/(m·K)	−2.401 × 10^−8^*T_a_*^2^ + 7.554 × 10^−5^*T_a_* + 2.364 × 10^−2^	[39]
Dynamic viscosity of hot air μ*_a_*/(Pa·s)	−3.238 × 10^−11^*T_a_*^2^ + 4.839 × 10^−8^*T_a_* + 1.73 × 10^−5^	[39]
Diffusion coefficient of moisture in hot air *D_a_* (m^2^/s)	3.229 × 10^−10^*T_a_*^2^ + 1.577 × 10^−7^*T_a_* + 2.089 × 10^−5^	[39]
Latent heat of vaporization $h_{g}$ (J/kg)	2,256,267	[40]

## Data Availability

Data are contained within the article.
